# The Spatio-Temporal Patterns of Regional Development in Shandong Province of China from 2012 to 2021 Based on Nighttime Light Remote Sensing

**DOI:** 10.3390/s23218728

**Published:** 2023-10-26

**Authors:** Hongli Zhang, Quanzhou Yu, Yujie Liu, Jie Jiang, Junjie Chen, Ruyun Liu

**Affiliations:** 1 School of Geography and Environment, Liaocheng University, Liaocheng 252059, China; zhang304279228@163.com (H.Z.); jiangjie1592020@163.com (J.J.); chenjunjie0103@163.com (J.C.); liuruyun0414@126.com (R.L.); 2 Yellow River Research Institute, Liaocheng University, Liaocheng 252000, China; 3 Research Center for Ecological Civilization, Chinese Research Academy of Environmental Sciences, Beijing 100012, China; liuyj@craes.org.cn

**Keywords:** nighttime light remote sensing, Shandong Province, regional development, difference analysis, temporal and spatial pattern

## Abstract

As a major coastal economic province in the east of China, it is of great significance to clarify the temporal and spatial patterns of regional development in Shandong Province in recent years to support regional high-quality development. Nightlight remote sensing data can reveal the spatio-temporal patterns of social and economic activities on a fine pixel scale. We based the nighttime light patterns at three spatial scales in three geographical regions on monthly nighttime light remote sensing data and social statistics. Different cities and different counties in Shandong Province in the last 10 years were studied by using the methods of trend analysis, stability analysis and correlation analysis. The results show that: (1) The nighttime light pattern was generally consistent with the spatial pattern of construction land. The nighttime light intensity of most urban, built-up areas showed an increasing trend, while the old urban areas of Qingdao and Yantai showed a weakening trend. (2) At the geographical unit scale, the total nighttime light in south-central Shandong was significantly higher than that in eastern and northwest Shandong, while the nighttime light growth rate in northwest Shandong was significantly highest. At the urban scale, Liaocheng had the highest nighttime light growth rate. At the county scale, the nighttime light growth rate of counties with a better economy was lower, while that of counties with a backward economy was higher. (3) The nighttime light growth was significantly correlated with Gross Domestic Product (GDP) and population growth, indicating that regional economic development and population growth were the main causes of nighttime light change.

## 1. Introduction

Under the background of high-quality economic development in China, the study of regional development differences has become a hot issue in the study of human geography. Fan et al. [1] analyzed the evolution of China’s regional development pattern since the reform and opening up and discussed the key points for the regulation and control of China’s regional development pattern in the next 30 years. Deng et al. [2] summarized the patterns of China’s regional balanced development in different periods, discussed the scientific connotation of regional balanced development and put forward some suggestions for China’s regional balanced development. Cao et al. [3] quantitatively measured the comprehensive development level of the Huaihe River economic belt based on the four dimensions, economy, society, ecological environment and sustainable development, and analyzed the spatio-temporal differentiation patterns and regional differences of regional development. Li studied the urban economic development of the Guangdong Province and put forward some feasible suggestions on how to solve the differences in regional social and economic development [4]. Entering the period of ‘The 14th five-year Plan’, China has begun a new journey of building a modern socialist country in every respect. In addition, people pay increasing attention to regional openness and inter-regional interaction. A regionally coordinated development strategy has been a dominant strategy to enhance and deepen inter-regional economic and social relations, which can solve the problem of imbalance and inadequacy in China’s economic development [5,6].

Most of these studies are based on the traditional methods of panel data analysis, which are highly dependent on statistical data [7]. On the one hand, statistical data are easily affected by people, and the accuracy and objectivity of them are affected. In addition, most of the statistical data are investigated by administrative units and lack more detailed analysis in space. As a special remote sensing image data, Nighttime Light data is closely related to patterns of urban economic development, which can comprehensively characterize the breadth and intensity of human activities. The recorded luminance value of light radiation has a unique effect in explaining the phenomenon of human activities and social and economic development, and can analyze economic and social activities on a relatively fine spatial scale [8]. For example, Meng estimated carbon emissions from 2000 to 2019 based on Defense Meteorological Satellite Program/Operational Linescan System (DMSP/OLS) Nighttime Light images of Jiangsu Province in China [9]. Using the Nighttime Light remote sensing time series data from 1992 to 2013, Martin quantitatively extracted and analyzed the information, and revealed the spatio-temporal patterns of China’s urbanization [10]. Zhang et al. [11] studied the variation of Nighttime Light in Pakistan from 2012 to 2019 at different geographical scales based on Visible Infrared Imager Radiometer Suite (VIIRS) monthly Nighttime Light remote sensing images. Ding et al. [12] studied the spatio-temporal patterns of county economic development in Shandong Province based on DMSP/OLS Nighttime Light data, using the method of spatial statistical analysis.

In the past 10 years, the economic and social development of Shandong Province has entered a new era, showing new patterns. There is an urgent need to clarify the temporal and spatial patterns and evolution laws of Shandong’s regional development, so as to provide a guarantee for the high-quality development of the Yellow River basin and realize the goal of “The peak of carbon emissions, carbon neutrality”. Therefore, aimed at the problem of regional development in Shandong Province, this study analyzes the distribution patterns and changing trend of Nighttime Light in Shandong Province by using pixel-based statistical analysis, trend analysis, stability analysis and spatial regional statistics and correlation analysis based on the monthly visible infrared imaging radiometer (National Polar-orbiting Partnership, Visible and Infrared Imaging Radiometer Suite, NPP/VIIRS) Nighttime Light remote sensing data from the past 10 years. From this perspective, clarifying the temporal and spatial patterns of the regional development of Shandong Province in the past 10 years allows us to provide some support for the high-quality development of Shandong Province.

With the rapid development of economic globalization and urbanization, cities are showing a trend for rapid development and competitive development, especially the development and growth of some large and medium-sized cities, which can not only be supported by national policies. In addition, they can absorb the resources of the surrounding areas and the scale of expansion is rapid. However, the development and expansion of many cities in the Shandong Province are not carried out according to the unified model, and each city has its own particularity. The form of urban spatial development will be affected by the direction and topography of the surrounding urban agglomeration integration, and will form its own unique development model. Since the beginning of new century, the external expansion and internal reconstruction of urban structures are the main patterns in China’s urban spatial development. Against this background, multi-core structures appear in big cities, commercial plots gather, and new commercial districts are gradually formed. Industrial development has gradually gathered into industrial zones and economic development zones, and urban space tends to be regionalized.

This study is of great significance to the research on the exploration of regional social and economic development based on Nighttime Light data, which can reflect the regional social and economic situation of Shandong Province to a considerable extent, and help to explore the law of temporal and spatial changes of social and economic activities. It can provide a scientific basis for relevant government departments to scientifically and reasonably formulate regional macro development strategy decisions, so as to quickly promote the coordinated development of urban agglomeration, and city and county integration in Shandong Province. It is beneficial for Shandong Province to become a demonstration area for the development of international urban agglomeration.

## 2. Data and Methods

### 2.1. General Situation of the Study Area

Shandong Province is located on the eastern coast of China and the lower reaches of the Yellow River. The province has a land area of about 155,800 km^2^ and a marine area of 159,600 km^2^. According to the seventh national census, the resident population of the province is 101.527 million. The regional GDP in 2021 was 8.30959 trillion ¥, an increase of 8.3% compared to the previous year. Shandong Province ranks third in GDP and second in population in the country. It is the largest economic and populous province in China. By the end of 2020, Shandong Province had jurisdiction over 16 prefecture-level cities, 136 county-level administrative districts (58 municipal districts, 26 county-level cities and 52 counties) and 1822 township-level administrative districts. Shandong Province belongs to the warm, temperate monsoon climate, with the same season of rain and heat, an annual average temperature of 11–14 (Celsius temperature) and an average annual precipitation of 550–590 mm [13]. The precipitation gradually decreases from the southeast coast to the northwest inland. The proportion of crop planting area is large; this is an important major grain producing area in China. The distribution characteristics of vegetation have obvious spatial differences. Shandong Province is a fast-growing economic province along the eastern coast of China, which plays an important role for the whole country. However, there are still issues of spatial imbalance and incoordination in the regional economic and social development of Shandong Province [14,15]. Shandong Peninsula urban agglomeration as a whole is more developed than central and western Shandong, and the development speed here is the fastest [12].

### 2.2. Data

#### 2.2.1. NPP/VIIRS Nighttime Light Data

This study used the monthly NPP/VIIRS (National Polar-orbiting Partnership, Visible and Infrared Imaging Radiometer Suite) Nighttime Light remote sensing data from April 2012 to October 2021. A total of 115 images were used. The data sensor, carried by the Suomi NPP satellite from the United States, was launched and put into use in 2011. Its specific parameters are shown in Table 1. The NPP/VIIRS Nighttime Light data unit is nW/cm²/sr. Before the monthly average image is released, the NPP/VIIRS data are processed by stray light, moonlight, cloud and other factors.

#### 2.2.2. Auxiliary Data for Regional Development Analysis

In addition, the study uses Shandong statistical yearbook data, such as GDP, population and annual carbon emissions from various prefecture-level cities in Shandong Province, as relevant indicators of regional development, and analyzes the correlation with Nighttime Light data. Meanwhile, it uses the boundary vector data of Shandong Province and its prefecture-level cities provided by the National basic Geographic Information Center [17], and the land use type data of Shandong Province provided by the Resource and Environmental Science and data Center of the Chinese Academy of Sciences [18]. The multi-source data used in this paper are publicly available, and the dataset information is shown in Table 2.

### 2.3. Methods

#### 2.3.1. Nighttime Light Data Preprocessing

**Figure 1:**
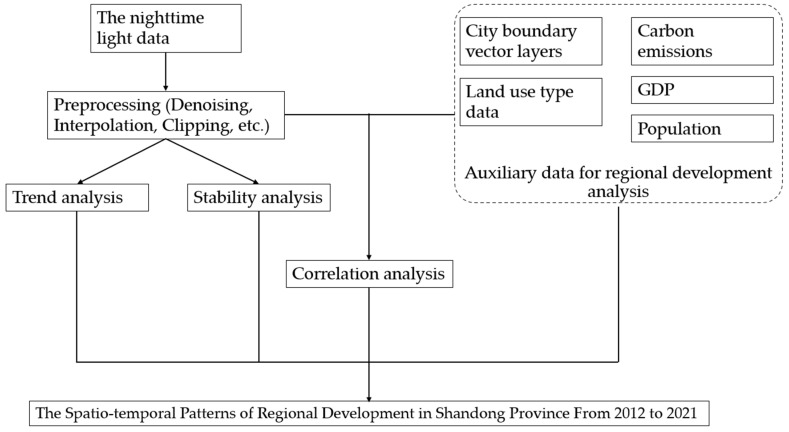
Data processing flow chart.

The noctilucent data preprocessing, downloaded from the EOG (Earth Observation Group) website, includes data denoising, data interpolation and projection conversion [16] to convert the processed image projection into an equal area projection coordinate system [20]. Due to the influence of stray light, the “vcmcfg” product of NPP/VIIRS data has a serious numerical deficiency in the middle and high latitudes of summer [21], and the officially revised “vcmslcfg” product still lacks June data for 2012 and 2013, so it is interpolated with the May and July averages of 2012 and 2013, respectively. After filling up the missing data, the vector boundary of Shandong Province is used to clip the monthly Nighttime Light images of Shandong Province. Data flow processing is shown in Figure 1.

#### 2.3.2. Statistical Analysis Based on Pixels

The univariate linear regression method was used to fit and analyze the changing trend. The DN value of Nighttime Light data and the change rate of related socio-economic elements are defined as the slope of a linear regression equation that changes month by month during the study period expressed by a slope; the formula is as follows:

(1) $$Slope=\frac{n*\sum_{i=1}^{n}\left(i*{DN}_{i}\right)-\sum_{i=1}^{n}i*\sum_{i=1}^{n}{DN}_{i}}{n*\sum_{i=1}^{n}t_{i}^{2}-{\left(\sum_{i=1}^{n}t_{i}\right)}^{2}}$$

In the formula, the variable *i* is the sequence number of the monthly synthetic image according to time, and the value of *i* is 1,2, …, 115; *n* is the number of samples, *n* = 115; *DN_i_* is the *DN* value of the *i* scene image; *t_i_* is the corresponding time of the *i* scene image (*t_i_* = YEARi + MONTHi/12, in which YEAR_i_ and MONTH_i_ are the year and month of the I scene image, respectively); and Slope is the slope of the regression equation during the study period, if slope > 0, it indicates that the Nighttime Light or some element index of the pixel in this period shows an upward trend, otherwise it decreases. The slope of the equation is regarded as the trend of Nighttime Light change.

For stability analysis of Nighttime Light change in the past 10 years, this study uses Formulas (2) and (3) to calculate the coefficient of variation of Nighttime Light value per pixel from 2012 to 2021, so as to reflect the degree of Nighttime Light fluctuation on different pixels in the study area in the past 10 years. The large fluctuation value indicates that the disturbance intensity is large and unstable; the small fluctuation value means that the state is stable.

(2) $$S=\sqrt{\frac{\sum_{i=1}^{n}{({DN}_{i}-\bar{DN)}}^{2}}{n-1}}$$

(3) $$Cv=\frac{S}{\bar{DN}}$$

In the formula: *Cv* is the coefficient of variation, *S* is the standard deviation, (*DN*) is the average value of *DN* for many years and the variable *i* is the sequence number of the monthly synthetic image according to time.

#### 2.3.3. Spatial Regional Statistics and Correlation Analysis

After downloading the data on land use types in Shandong Province in 2020, the built area is extracted to study the statistical patterns and changes of Nighttime Light in Shandong Province over the past 10 years. The land use types in Shandong Province in 2020 are shown in Figure 1. Pearson correlation analysis was carried out between the Nighttime Light statistics and the corresponding data for GDP, population and carbon emissions in each city and built-up area, in order to make clear the reason for the change in Nighttime Light and its effect in indicating regional development.

## 3. Results and Discussion

### 3.1. Spatio-Temporal Pattern of Nighttime Light in Shandong Province

#### 3.1.1. Present Situation of Land Use and Average Nighttime Light Pattern

**Figure 2:**
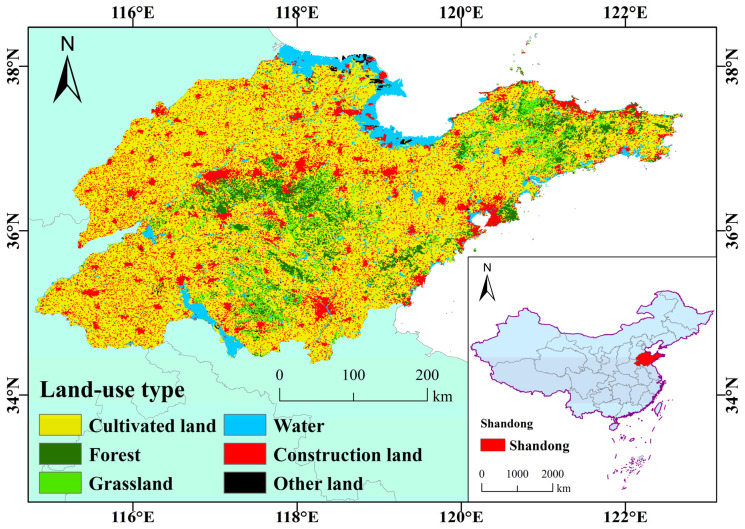
Land cover in Shandong province in 2020.

**Figure 3:**
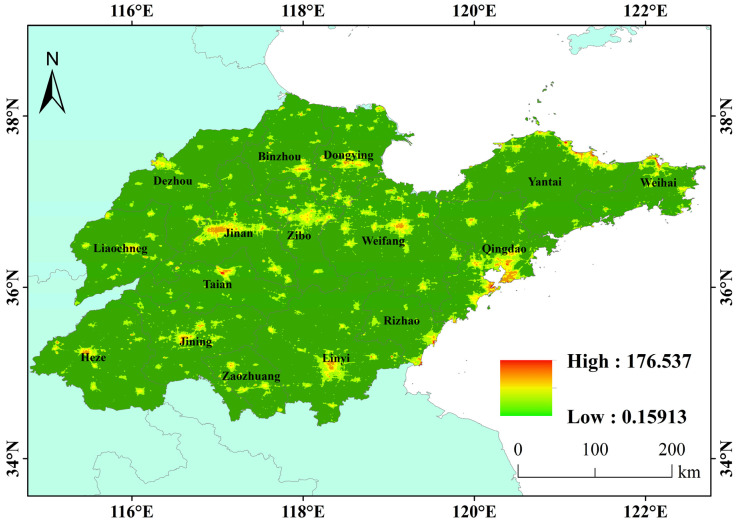
Spatial pattern of mean Nighttime Light in Shandong province from April 2012 to October 2021.

Figure 2 and Figure 3 show that in the past 10 years, there has been obvious spatial consistency between the Nighttime Light mean distribution and the land use pattern in Shandong Province, especially with the urban construction land distribution. The areas with higher lighting at night are concentrated in the area of construction land, and emphasize a trend of gradually decreasing from the center to the edge of the construction land. In particular, it shows the patterns of the bipolar leadership of Qingdao and Jinan, and the multi-point scattered development of Weifang, Linyi, Zibo and Yantai. As far as each prefecture-level city is concerned, it shows the distribution patterns of one core plus multiple points, and the lighting has a good consistency with the resident distribution pattern of cities and counties. The Nighttime Light of other land types is generally low.

In terms of Nighttime Light intensity, the Nighttime Light intensity of prefecture-level cities is significantly higher than that of county-level cities, and that of the main urban areas is higher than that of suburbs and rural areas. The coastal Nighttime Light is higher than inland; the coastal areas of Shandong Peninsula are economically developed, and the Nighttime Light of towns here is generally high. At the same time, Dongying oil field and coastal ports have large-scale infrastructure and continuous operation, and the Nighttime Light brightness is also significant. Nighttime light is mainly distributed in economic and densely populated spot areas such as Jinan and Qingdao, but also shows a zonal distribution along the traffic line and coastline.

#### 3.1.2. Spatio-Temporal Variation in Nighttime Lights in the Past Ten Years

**Figure 4:**
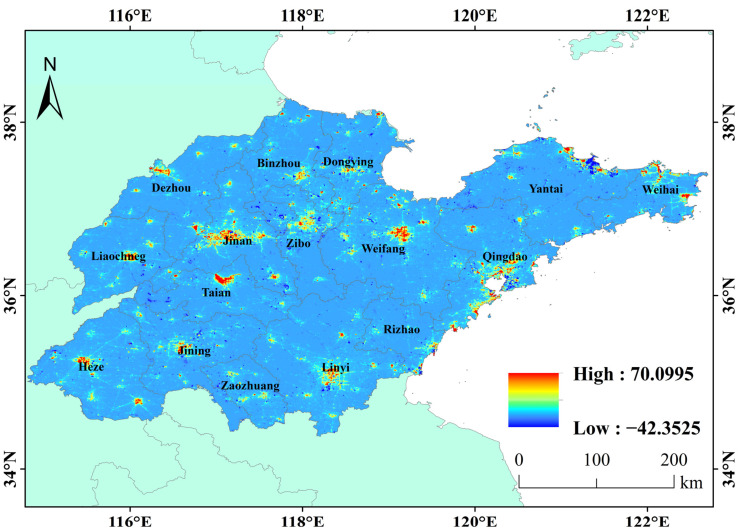
Spatio-temporal changes in Nighttime Light in Shandong province from April 2012 to October 2021.

**Figure 5:**
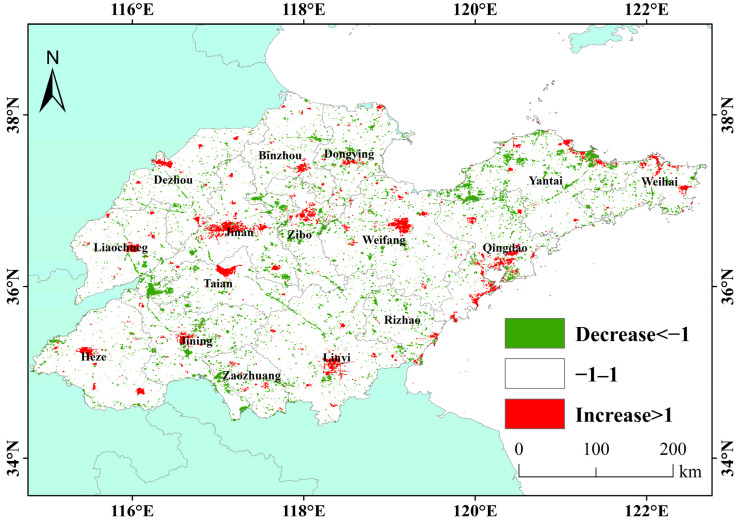
Key areas of Nighttime Light change in Shandong province from April 2012 to October 2021.

The changing trends in Nighttime Light can be seen from Figure 4 and Figure 5. Figure 4 displays the slope of Nighttime Light growth over the past decade. Red represents a rapid increase and blue represents a decrease. In Figure 5, red means more than 100% compared to its own growth, green means a reduction of 100%. The Nighttime Light changes slowly in most areas in Shandong Province, but there is also obvious spatial heterogeneity of Nighttime Light changes in some areas. The changing areas are mainly distributed in the coastal areas of the Shandong Peninsula, along the Jinan-Weifang section of Jiaoji Railway, along the Shandong section of the east line of The South-to-North Water Diversion Project and in urban areas in western Shandong. Specifically, the administrative stations of most prefecture-level cities and some surrounding counties have an obvious increasing trend in Nighttime Light, and the intensity of growth weakens from the city center to the periphery, indicating that, in the past 10 years, urbanization has developed rapidly, and the urban infrastructure has been gradually improved. However, the Nighttime Light in Qingdao, Yantai and Weihai in Shandong Peninsula has unique patterns. The Nighttime Light in the core urban areas decreases or does not obviously change, while the Nighttime Light in the new urban areas around it clearly increases. This feature is most obvious in Qingdao, where there is a downward trend in Nighttime Light in the old urban area of the east coast, and there is an obvious growth trend in the new area of the west coast. This may also imply that the urban economy of Jiaodong Peninsula has entered a stage of high-quality development.

The decline in Nighttime Light in Taian City is mainly in Dongping County, especially near Dongping Lake; there is a similar decline near Nansi Lake in Jining. This may be due to the local closure of many industrial and mining enterprises in order to prevent the impact of pollution on the fishery of Dongping Lake [22], and the establishment of closed fishing periods and areas in the lake area to prevent overfishing [23]. At the same time, the county government actively guided fishermen to return to the lake and to change production, but a large number of fishermen joined other industries [24]. Zaozhuang is listed as one of the second batch of resource-exhausted city transformation pilot cities under the State Council, and the reduction in coal mine output has to some extent affected the Nighttime Light [25,26]. Jining is one of the thirteen coal energy bases in China, and the local coal mining subsidence is controlled according to local conditions [25]. This may also lead to a downward trend in its Nighttime Light.

**Figure 6:**
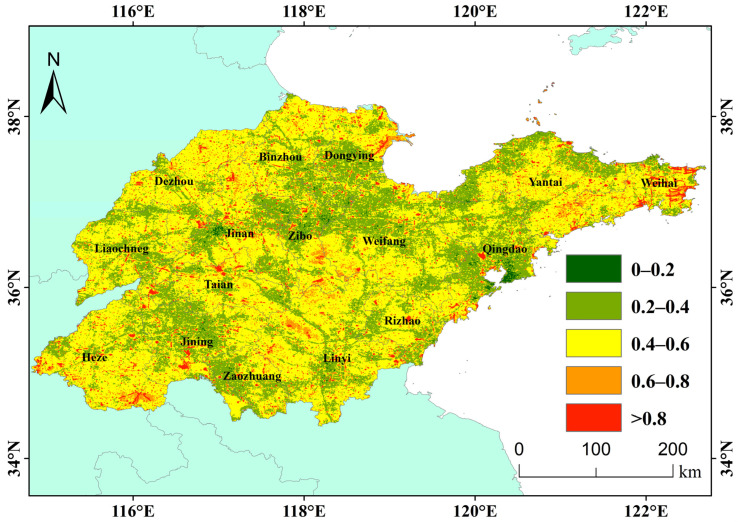
Stability pattern of Nighttime Light in Shandong province from April 2012 to October 2021.

We divide the Nighttime light stability level into five levels, as shown in Figure 6; this picture shows stability, which means the absolute value of change. The most stable area of Nighttime Light in Shandong Province in the past 10 years (*Cv* < 0.2) is located in the old urban areas of Qingdao and Jinan, indicating that the changes in urban lighting in the old urban areas of big cities have been relatively gentle in the past 10 years, and the infrastructure in the old urban areas may not have changed significantly, or it may imply that the relationship between deep economic development and Nighttime Light growth is not significant. The most unstable areas (*Cv* > 0.8) are scattered across the whole province, among which the more concentrated areas are the central and eastern part of Weihai, the estuary of the Yellow River Delta, the provincial capital, metropolitan area and the periphery of related cities in southern Shandong and the north of Jiaozhou Bay in Qingdao. On the one hand, the instability of Nighttime Light in these areas may be due to drastic changes in infrastructure in the periphery of cities due to the intensification and expansion of cities. On the other hand, the construction and operation of new infrastructure may also lead to Nighttime Light instability in these areas. For example, the construction of Qingdao Jiaodong International Airport [27], the traffic trunk road, the construction of oil industry facilities in the Yellow River Delta [28] and the construction of Huangdao and the Huangdao national oil storage caverns [29]. In short, drastic changes in land use, especially those related to infrastructure land, lead to drastic changes in Nighttime Light.

### 3.2. Nighttime Light Differences on Different Spatial Scales

#### 3.2.1. Nighttime Light Differences of Three Geographic Units in Shandong Province

According to previous data, Shandong Province is divided into three geographical regions: northwest Shandong, south-central Shandong and eastern Shandong [30]. The northwest of Shandong includes Dongying, Binzhou, Dezhou, Liaocheng, Heze, Liangshan County, Wenshang County, Jiaxiang County, Jinxiang County, Yutai County, Weishan County and Liangshan County in Jining, as well as Shouguang City in Weifang, Gaoqing County in Zibo, Dongping County in Taian, Jiyang District and Shanghe County in Jinan. South-central Shandong includes all counties in Jinan except Jiyang District and Shanghe County, all of Zibo County except Gaoqing County, all of Weifang County except Shouguang City, Gaomi City and Zhucheng City, all of Tai’an County except Dongping County, the Jining City area, Zoucheng City, Qufu City, Sishui County, Zaozhuang and Linyi. Eastern Shandong mainly includes Weihai, Yantai, Qingdao, Rizhao and Gaomi City and cities in Weifang.

In Table 3 it can be seen that the total amount of Nighttime Light in each region has increased significantly from 2012 to 2021, which means that the overall economy of Shandong Province has developed to a great extent in these 10 years. Among them, the Nighttime Light growth rate in the northwest of Shandong is the highest, reaching 245.95%, which is almost twice that of the eastern Shandong. The Nighttime Light growth rate in the central and southern parts of Shandong is in the middle, and the Nighttime Light growth rate gradually increases from the eastern Shandong through central and southern Shandong to the northwest of Shandong. It shows that, although the level of economic development in northwest Shandong is relatively backward, the society has developed rapidly in recent years, and even the degree of relative growth in this decade has greatly exceeded that of the economically developed eastern Shandong region. At the same time, it also implies that the economic development stages of the three major geographical units in Shandong Province are not synchronized, that is, they are in different stages of high-quality development; the development of northwest Shandong tends towards the traditional extensive economic development model. The consumption of energy and electricity may be greater, while the development of eastern Shandong tends towards an intensive and high-quality development model.

#### 3.2.2. Nighttime Light Patterns at the Urban Scale in Shandong Province

According to Table 4, the total amount of Nighttime Light in all prefecture-level cities in Shandong Province showed an increasing trend from 2012 to 2021. Liaocheng had the largest Nighttime Light growth rate of 309.54%, followed by Dezhou City (199.20%), Linyi City (191.27%), Heze City (186.45%) and so on. Although the level of economic development of Liaocheng, Dezhou and Heze is relatively backward in Shandong Province, they have grown rapidly in the past 10 years, indicating that the relative development of these three cities is very fast, and the gap between these three cities and developed cities is narrowing. The cities with the lowest increase in total Nighttime Light volume from 2012 to 2021 were Yantai (80.02%), Zibo (91.17%), Dongying (92.82%) and Jinan (95.57%). The level of social development in these cities is relatively high, and the dependence of their economic development on resources and electricity may be relatively low, so the lighting growth rate is relatively low.

Nearly 10 years of Nighttime Light growth slope (slope) data show that the urban Nighttime Light total slope is the highest in Qingdao, Jinan and Weifang, while Linyi and Yantai’s Nighttime Light slopes are negative. On the one hand, this may be related to the overall economic development model and speed of the city, and it also has a lot to do with the degree of coordinated development in urban and rural areas. The total value of the growth slopes for urban and rural areas with well coordinated development is generally higher.

#### 3.2.3. Nighttime Light Patterns at Counties Scale in Shandong Province

There are 136 districts and counties in Shandong Province, and they are ranked according to their GDP; Table 5 shows the top five districts in terms of GDP. The total amount of GDP and the Nighttime Light slope in Jinan is far ahead than the rest of the province. But in terms of nighttime light growth rate, Huangdao District is far higher than other areas, which was 163.57%. The growth rate and change in the Yantai district are ranked low among the five districts and counties. The districts and counties in the bottom five in terms of GDP are shown in Table 6. As can be seen from Table 6, the total amount of Nighttime Light in Chengwu County in Heze increased rapidly from 2012 to 2021, with a growth rate of 229.18%. This was followed by Gaotang County in Liaocheng (167.25%), Qingyun County in Dezhou (156.73%), Zhanhua County in Binzhou (95.44%) and Dong’e County in Liaocheng (81.51%). The total-amount-of-Nighttime-Light slope also proves this point; the change rate for total Nighttime Light amount corresponds to the Nighttime Light slope, and the districts and counties with high rates of change in Nighttime Light area also have high Nighttime Light slopes. Overall, the Nighttime Light growth rate of economically backward districts and counties is relatively high, while that of better economic districts and counties is the opposite.

### 3.3. Correlation Analysis of Light Change at Nighttime Light

#### 3.3.1. The Relationship between the Total Amount of Nighttime Light and Economic Indicators in Various Prefecture-Level Cities

Using the built-up area range of land use extraction in Shandong Province in 2020, bivariate Pearson correlation analysis was carried out between the mean Nighttime Light for each city region and urban core area from April 2012 to October 2021 and the mean ten-year GDP value, the ten-year population average value and the average carbon emission value for each city (six years’ worth of data from 2012 to 2017). The results are shown in Table 7.

It is found that there is a significant correlation between the mean Nighttime Light intensity and GDP in both the urban unit and the urban core area, especially in the core area. There is a very significant positive correlation between the Nighttime Light mean and its GDP (*p* < 0.01), indicating that the Nighttime Light intensity in the core urban area is closely related to economic development. The correlation between average Nighttime Light and population and carbon emissions is not significant; however,, it may imply that the increase in population can not be directly reflected in energy and electricity consumption. In addition, carbon emission data may lead to insignificant patterns due to time scale mismatch. In short, it can be explained that the average Nighttime Light value can be used as an effective index to measure regional economic development.

#### 3.3.2. The Correlation between Nighttime Light Growth and Economic Index Growth in Cities at Each Prefecture Level

In order to further analyze the relationship between the Nighttime Light index and regional development, the relationship between Nighttime Light index growth and economic index growth is studied and calculated. Based on the annual growth (slope) of mean Nighttime Light from April 2012 to October 2021 in 16 prefecture-level cities in Shandong Province, and the slope of GDP, the slope of population and the slope of carbon emissions (2012–2017), the Pearson correlation is calculated. It can be seen from Table 8 that there is a significant positive correlation between the Nighttime Light slope and slope of GDP in 16 prefecture-level cities, and there is also a significant correlation between the urban Nighttime Light slope and the population slope, indicating that the growth of urban Nighttime Light intensity is closely related to local human, social and economic activities.

However, the Nighttime Light slope in the core areas of 16 prefecture-level cities was not correlated with the slope of population, the slope and carbon emission or the slope of GDP. Further comparing the data in the table, it is found that the correlation between the GDP slope and the population slope has decreased, indicating that the social and economic activities in urban non-core areas (rural areas) of 16 prefecture-level cities in Shandong Province are also factors affecting Nighttime Light slope.

### 3.4. Limitations and Prospects

Although this study has made some breakthroughs in describing the regional devel-opment pattern of Shandong Province, due to the limitations of data acquisition and workload, there are still some shortcomings. The Night Light remote sensing sensor used was disturbed by cloud, fog, terrain and other factors in the imaging process, and the available multi-temporal data in the study area is limited. The method models used are too few and have not been cross verified with other remote sensing data.

The spatio-temporal resolution is not fine enough and can be analyzed by combining a variety of Nighttime Light satellites in the future. 

At the same time, Nighttime Light remote sensing data can not only be combined with GDP and population data to study urban economic development, but also can be used for the urban belt and urban agglomeration development trajectory monitoring, urban land use scale prediction, poverty index and other related research. 

Models can be built to make Nighttime Light data better reflect regional economic activities.

## 4. Conclusions

In this study, the spatio-temporal variation patterns and reasons for Nighttime Light in Shandong Province are analyzed on the basis of three geographical units, city scale, county scale and pixel scale. There were different regional development models used on different scales, among which the most obvious one is at the city scale. The main results are as follows: 

(1) The average Nighttime Light in Shandong Province in the past 10 years decreases from the center of the urban built-up area to the edge. From the spatio-temporal patterns of provincial Nighttime Light, the average Nighttime Light distribution is consistent with that of construction land. In the past 10 years, the change pattern for Nighttime Light showed a certain spatial heterogeneity, the Nighttime Light in the built-up areas of most cities showed an increasing trend, while in the old urban areas of Qingdao and Yantai and other non-urban areas, the Nighttime Light showed a weakening trend. This may be related to different stages of regional social development.

(2) There are differences in the total Nighttime Light amount and mean value of the three geographical units in Shandong Province, and the Nighttime Light growth rate is higher in the northwest of Shandong Province (246%), which has a lower level of economic development. The growth rate of Nighttime Light in eastern Shandong, which has a higher level of economic development, is lower. The Nighttime Light growth rate increases sequentially from the eastern part of Shandong through the central and southern parts of Shandong and into the northwest of Shandong. It implies that the level of regional development in eastern Shandong, south-central Shandong and northwest Shandong is becoming increasingly balanced.

(3) At the urban scale, Nighttime Light growth does not completely match its level of economic development. The economic development level of Liaocheng, Dezhou and Heze is relatively backward, but Nighttime Light has increased rapidly in the past 10 years, by 309.51%, 199.20% and 186.45%. While the peninsula cities, such as Yantai and Qingdao, have a high economic output, the Nighttime Light growth is slow, at 80.02% and 125.61%, and some built-up areas have declined. At the same time, in areas like Zaozhuang City, the growth rate of Nighttime Light is slow due to the influence of the South-to-North Water Diversion Project and the reduction in local coal mine production. This shows that different cities may be in different stages of regional high-quality development.

(4) At the county and district scale, except in Huangdao District, the Nighttime Light growth rate of the top five counties in 2020 is relatively slow, especially in Yantai City. The Nighttime Light growth rate of the counties in the bottom five in 2020 is relatively high, especially in Chengwu County in Heze. This implies that the regional consumption of resources and electricity is different at different stages of economic development.

(5) The correlation analysis at the city level showed that there was a significant positive correlation between the mean Nighttime Light and GDP (R = 0.60, *p* < 0.05), especially in the urban area (R = 0.60, *p* < 0.01). In terms of growth, there is a significant correlation between Nighttime Light growth and GDP and population growth (*p* < 0.05). This shows that the main factors affecting Nighttime Light growth are economic development and population growth, and it also shows that Nighttime Light data can better reflect regional development.

## Figures and Tables

**Table 1:** Satellite and sensor parameters involved [16].

Data Type	NPP/VIIRS
Strip width	~3000 km
Night transit time (local time)	~01:30
Low light imaging bandpass	Panchromatic 0.5–0.9 μm
Value	Absolute radiation value
Spatial resolution	15 arc seconds (500 m)
Calibration	Satellite calibration
Saturation	None
Product cycle	Month
Time series	Daily data: 20120201 to present
	Monthly composite data: 201204 to present

**Table 2:** Auxiliary data used in this study.

Data Name	Year	Source	Resolution
GDP	2020	Shandong statistical yearbook	
Population	2020	Shandong statistical yearbook	
Carbon emissions	2020	Shandong statistical yearbook	
City boundary vector layers	2020	National basic Geographic Information Center	
Land use type data	2020	Resource and Environmental Science and data Center of the Chinese Academy of Sciences	30 M
NPP-VIIRS of monthly	April 2012–October 2021	Earth Observation Group [19]	500 M

**Table 3:** Nighttime Light change data from three geographical units in Shandong province from April 2012 to October 2021.

Region	Total Amount of Regional Nighttime Light/(nW/cm²/sr)		Nighttime-Light Growth-Scale /(nW/cm²/sr)	Nighttime Light Growth Rate /%	Total Amount of Nighttime Light Slope/(nW/cm²/sr)
	Year 2012	Year 2021			
Northwest Shandong	173,355.01	599,725.90	426,370.89	245.95%	16,055.57
South-central Shandong	311,984.31	781,625.68	469,641.37	150.53%	6204.76
Eastern Shandong	208,258.41	467,140.42	258,882.01	124.31%	4655.77
Total	693,597.73	1,848,492.00	1,154,894.27	166.51%	26,916.10

**Table 4:** Nighttime Light change data of 16 prefecture-level cities in Shandong province from April 2012 to October 2021.

Region	Total Amount of Regional Nighttime Light/(nW/cm²/sr)		Nighttime-Light Growth-Scale/(nW/cm²/sr)	Nighttime Light Growth Rate/%	Total Amount of Nighttime Light Slope/(nW/cm²/sr)
	Year 2012	Year 2021			
Binzhou	44,650.55	105,405.01	60,754.47	136.07%	3930.26
Dongying	52,951.51	102,103.42	49,151.92	92.82%	3281.23
Dezhou	32,894.84	98,421.34	65,526.50	199.20%	6172.03
Heze	49,310.93	141,253.51	91,942.58	186.45%	6581.39
Jinan	104,759.04	204,873.39	100,114.35	95.57%	9012.95
Jining	55,779.22	142,984.11	87,204.89	156.34%	3524.73
Liaocheng	24,811.57	101,606.38	76,794.81	309.51%	5025.86
Linyi	59,740.39	174,008.03	114,267.65	191.27%	−397.83
Qingdao	113,954.34	257,093.59	143,139.25	125.61%	11,365.05
Rizhao	24,306.46	56,928.44	32,621.99	134.21%	1855.87
Taian	32,926.04	84,017.92	51,091.88	155.17%	3225.36
Weifang	85,184.51	198,850.53	113,666.02	133.44%	8814.29
Weihai	29,399.35	78,879.87	49,480.52	168.30%	5310.10
Yantai	80,574.92	145,051.16	64,476.25	80.02%	−207.45
Zibo	55,354.61	105,823.48	50,468.88	91.17%	4229.70
Zaozhuang	27,161.18	60,815.65	33,654.47	123.91%	2186.87
Total	873,759.46	205,8115.86	1184,356.40	135.55%	73,910.42

**Table 5:** Data for Nighttime Light changes in the top five economic districts and counties of Shandong Province from April 2012 to October 2021.

Region	GDP in 2020/RMB100 Million	Total Amount of Regional Nighttime Light/(nW/cm²/sr)		Nighttime-Light Growth-Scale/(nW/cm²/sr)	Nighttime Light Growth Rate/%	Total Amount of Nighttime Light Slope/(nW/cm²/sr)
		Year 2012	Year 2021			
Central urban area of Jinan	7533.1	57,930.91	112,657.13	54,726.22	94.47%	5522.16
Central urban area of Qingdao	4876.4	53,441.99	88,023.37	34,581.37	64.71%	2150.36
Huangdao District	3721.7	15,614.12	41,153.98	25,539.86	163.57%	2599.66
Central urban area of Yantai	3467.3	24,491.77	34,625.67	10,133.90	41.38%	284.05
Central urban area of Zibo	2633.7	41,671.64	72,272.46	30,600.82	73.43%	2406.66
Total	22,232.2	193,150.44	348,732.61	155,582.17	80.55%	12,962.88

**Table 6:** Nighttime Light change data of five districts and counties in Shandong Province from April 2012 to October 2021.

Region	GDP in 2020/RMB100 Million	Total Amount of Regional Nighttime Light/(nW/cm²/sr)		Nighttime-Light Growth-Scale/(nW/cm²/sr)	Nighttime Light Growth Rate/%	Total Amount of Nighttime Light Slope/(nW/cm²/sr)
		Year 2012	Year 2021			
Dong’e County	147.2	4185.12	7596.63	3411.50	81.51%	141.27
Gaotang County	151.2	3194.49	8537.23	5342.74	167.25%	477.22
Chengwu County	155.4	2407.41	7924.80	5517.39	229.18%	527.43
Zhanhua County	162.6	4384.32	8568.94	4184.61	95.44%	269.07
Qingyun County	165.5	1566.77	4022.39	2455.62	156.73%	282.32
Total	781.9	15,738.12	36,649.98	20,911.86	132.87%	1697.32

**Table 7:** Correlation analysis between ten-year Nighttime Light average and GDP, population and carbon emissions in Shandong province.

		Average GDP	Average Population	Average Carbon Emission
The average Nighttime Light value of each prefecture-level city	Pearson correlation	0.600 *	0.350	0.379
	Significant (double tail)	0.014	0.184	0.148
	Number of cases	16	16	16
Average Nighttime Light value of urban core area	Pearson correlation	0.748 **	0.013	0.403
	Significant (double tail)	0.001	0.963	0.122
	Number of cases	16	16	16

* at level 0.05 (double tail), the correlation was significant. ** at level 0.01 (double tail), the correlation was significant.

**Table 8:** Correlation analysis between nighttime light emission and slope of GDP, population and carbon emissions in Shandong.

		Slope of GDP	Slope of Population	Slope of Carbon Emission
Urban Nighttime Light slope	Pearson correlation	0.570 *	0.531 *	~0.177
	Significant (double tail)	0.021	0.034	0.513
	Number of cases	16	16	16
Nighttime Light slope in the core area of the city	Pearson correlation	0.45	0.378	~0.185
	Significant (double tail)	0.08	0.149	0.493
	Number of cases	16	16	16

* at level 0.05 (double tail), the correlation was significant.

## Data Availability

The multi-source data used in this paper are publicly available, and the dataset information is shown in Table 2.
